# Adolescent offenders' current whereabouts predict locations of their future crimes

**DOI:** 10.1371/journal.pone.0210733

**Published:** 2019-01-30

**Authors:** Wim Bernasco

**Affiliations:** 1 Netherlands Institute for the Study of Crime and Law Enforcement (NSCR), Amsterdam, the Netherlands; 2 Department of Spatial Economics, School of Business and Economics, Vrije Universiteit Amsterdam, Amsterdam, the Netherlands; Victoria University of Wellington, NEW ZEALAND

## Abstract

Knowing where crime is likely to happen can help prevent it. Here I investigate whether two basic mechanisms of human mobility—preferential return and spatial exploration—explain and predict where offenders commit future crimes. A sample of 843 adolescents reported their hourly whereabouts during four days. In line with findings from other sources and populations, their locations were concentrated and predictable. During the subsequent four years, 70 of the 843 were apprehended for committing one or more crimes. Compared to others, these 70 future offenders had visited slightly more different locations. However, their action radius and the predictability of their whereabouts had been very similar to those who would not become offenders. The offenders perpetrated most of their crimes around places they had visited before, including places where they previously offended. These findings show that the predictability of human mobility applies to offending and to offenders as well, and helps us understand and forecast where they will commit future crimes. They may prove particularly useful in criminal investigations, as they suggest that police should generally prioritize suspects who are familiar with the location of the crime and its environs, either because of their legal routine activities or because of their prior offences.

## Introduction

The prevention of crime heavily depends on our ability to forecast where and when it will happen. Recently, police forces have successfully started to allocate their resources using algorithms that predict where and when crime is most likely to occur next [1, 2]. The algorithms are based on well-corroborated evidence that crime risk is temporarily elevated within a few weeks and within a few hundred meters from a previous crime [3]. This space-time pattern of crime (but not its spatial and temporal scale) is similar to the patterns that characterize the diffusion of infectious diseases and the propagation of earthquakes. In fact, it has first been demonstrated with methods originally developed in epidemiology [4, 5] and in seismology [6].

Epidemiological and seismological models, however, do not reveal the underlying causes of crime patterns. People commit crimes, and analogies with viruses or tectonic forces hide the mechanisms that cause these patterns. To improve not only the prediction but also the explanation of where crimes take place, we need to understand the spatial behavior of individual offenders.

Crime pattern theory is a non-formalized theory that addresses the question where offenders perpetrate crime [7]. One of its key concepts is *activity space*, the area of an individual’s recurrent mobility. More specifically, it is defined as the set of locations where an individual routinely spends nontrivial amounts of time and the paths taken between these locations. The theory asserts that most crimes occur while the offenders are involved in non-criminal routine activities in or near their activity space. In addition, it proposes that even crimes not perpetrated during the offenders’ routine activities, are informed by what offenders heard, saw, read or picked up otherwise in the course of their daily routines.

In recent years, our knowledge of human spatial behavior has developed rapidly. This development is mainly due to the advent of mobile phones with geo-positioning functions that have made available large amounts of unobtrusively collected individual data on mobility [8–10].

Initially, and in line with findings on foraging patterns of human hunter-gatherers [11] and those of other species [12, 13], Lévy flight models were used to describe general human mobility [14]. Lévy flights have also been proposed as optimal target search strategies for offenders, as they would maximize encounter rates of suitable targets and minimize the risk of detection by law enforcement [15]. However, research on the whereabouts of mobile phone users has demonstrated that their spatial patterns strongly deviate from the randomness inherent in Lévy flights [10, 16]. Instead, and confirming prior findings on repetition and variability in travel behavior [17, 18], most human mobility follows cyclical daily and weekly routines, and is therefore highly predictable [8, 19]. Recent studies have proposed and confirmed the exploration and preferential return (EPR) model [16, 20–23] that comprises two simple mechanisms to explain and predict individual human mobility. The first mechanism is *preferential return*, the propensity to return to locations frequently visited before. The second mechanism is *spatial exploration*, the tendency to visit new locations nearby familiar ones.

Whereas preferential return and spatial exploration explain individual mobility patterns during routine activities, it is unknown whether they also apply to criminal conduct. Committing crime is a specific non-routine act that is comparatively rare even amongst notorious offenders. Therefore, the key assertion of crime pattern theory—that most crimes are committed in or near the offender’s activity space—still needs to be addressed. Empirical research using registry data suggests that preferential return and spatial exploration do also govern crime location choices, as offenders tend to perpetrate crimes near their homes [24], their previous homes [25], the homes of relatives [26] and places where they offended in the past [25, 27]. This body of evidence is tentative, however, as it is based on assumptions about where offenders spend time rather than on observations of their actual whereabouts.

Detailed observations of offender mobility are very rare. Only two prior small-scale studies used detailed geographic tracks of offenders during legal activities before their involvement in crime. One used global positioning system (GPS) data of 17 parolees who committed a crime while participating in an electronic monitoring program. It visualizes and describes the movement patterns of offenders before, during and after offending (26). The other study used mobile phone data records of terrorists during the months preceding their attacks. It examined how frequent the offenders visited various locations, and how far they traveled from key anchor points (27). Both studies utilized small and specific convenience samples, and neither study systematically investigated whether the routine mobility of the offenders could explain and predict where they would subsequently commit crimes.

Based on data from a larger and generic sample, the main purpose of the present study is to assess whether preferential return and spatial exploration can explain and predict where offenders will commit crimes in the future, even if these future crimes are infrequent and occur months or even years ahead. To this aim, I use the observed daily mobility patterns of a set of prospective offenders to predict the locations of their future crimes. A supplementary objective is to assess whether the routine mobility patterns of offenders differ from those of non-offenders.

## Hypotheses

The statistical analyses are informed and structured by the *crime pattern theory* [7]. The theory asserts that offenders perpetrate crimes in and nearby their activity spaces, locations they are already familiar with through their recurrent mobility. In other words, it proposes that preferential return and spatial exploration not only drive routine activities, but also crime location choices.

Two concrete hypotheses follow from the mechanism of preferential return. The *activity space* hypothesis (H1) states that offenders are more likely to commit crimes in their activity space than elsewhere. As suggested by the properties of the EPR-model [20], the *usage* hypothesis (H2) states that offenders are more likely to commit crimes in frequently used locations of their activity space than in locations they visit less often.

Further, it is suggested that offenders tend to explore areas immediately surrounding their activity space, typically one or two blocks off a known path [28]. Thus, according to the *spatial exploration* hypothesis (H3), a target is more likely located nearby than far away from the offender’s activity space.

Past crimes locations may play an important role in future crimes, because prior crimes create a template for future crimes. The *repeat location* hypothesis (H4) states that offenders are more likely to offend at the locations of their previous crimes than elsewhere, while the *near repeat location* hypothesis (H5) states that this also holds true for places nearby the previous crime location.

Crime pattern theory further emphasizes that the presence of criminal opportunity is a necessary requirement for crime, as without a suitable target crime will not take place. Criminal opportunities most frequently occur in locations where many potential victims converge and exchange valuable goods and services. The *opportunity* hypothesis (H6) states that locations with businesses and other points of interest create opportunities for crime and are therefore more likely to be chosen as crime sites than locations without such facilities.

## Materials and methods

The research was approved by the Ethics Committee for Legal and Criminological Research of the Faculty of Law of the Vrije Universiteit Amsterdam (CERCO).

### Sample

Details of the sampling procedure have been reported extensively elsewhere [29, 30] and are summarized here. The Study of Peers, Activities and Neighborhoods (SPAN) is a two-wave panel study among adolescents in The Hague, the Netherlands, and its suburbs. Data collection of the first wave took place between October 2008 and May 2009. Data collection of the second wave took place between November 2010 and June 2011. The study included a self-report questionnaire and a personal space-time budget interview, both of which were administered at the schools that the respondents attended. In the second wave, if respondents had left school, the interviews were held at other semi-public locations. All instruments were based on the materials of the Peterborough Adolescent and Young Adult Development Study (PADS) [31, 32]. In the present analysis, only the space-time budget data collected in the first wave is used, because this maximizes the time period over which offences could be observed that were perpetrated *after* the space-time budget interviews in which activity space was reported.

In the first wave of the study, 40 schools for secondary education were approached, of which 10 agreed to participate in the study, allowing students to participate in the study during school hours. The main reasons why schools refused to participate in the study were ongoing or recent participation in other research, and concerns about interference with regular school activities. The parents of the students were informed about the study and could easily refuse participation in both waves (passive consent).

The sample was designed to be conducted among a cohort of 1st graders (ages 12–13) and a cohort 4th graders (ages 15–16). In the second wave, two years later, most members of the younger cohort were in 3rd grade, while those of the older cohort were either in 6th grade of secondary education, in the 1st or 2nd grade of follow-up education, employed or jobless).

The sample was representative for adolescents in The Hague of these age groups in terms of gender and ethnic background. However, due to the selection of the 10 schools it contained an overrepresentation of adolescents enrolled in schools preparing for lower-level educational qualifications (18% against 15% in the population) and higher-level educational qualifications (23% against 19% in the population) and an underrepresentation of the intermediate group (59% against 66% in the population). Reference data for The Hague were calculated from data accessed at website https://denhaag.buurtmonitor.nl.

In total, 843 pupils participated in the first wave and 615 (73%) participated again in the second wave. The main reason for the 27% attrition rate is that students who had left the school they attended in the first wave were difficult to contact. Nonresponse analysis shows that boys, older and more delinquent respondents have somewhat higher attrition rates.

At the end of the second wave questionnaire, respondents were informed that the researchers were interested in collecting information about them from authorities like school, municipality and police, and they were asked for their written consent. Of the 615 that participated in the second wave, 517 (84%) gave their consent.

### Space-time budget interview

A space-time budget interview is a structured face-to-face interview conducted by a trained research assistant. I used a translated version of the materials developed in the Peterborough Adolescent and Young Adult Development Study (PADS+) The interviews took place during weekdays and involved the reconstruction of all major activities and whereabouts of the adolescents over four recent days, with a Friday and Saturday always being included, and Sundays always being excluded (e.g., interviews on Monday would cover the previous Wednesday to Saturday, interviews on Wednesday would cover the previous Friday, Saturday, Monday and Tuesday). Relative to the reference proportion of 1/6 = .167, the day-of-week selection procedure generates an overrepresentation of Fridays (.249) and Saturdays (.250) and underrepresentation of Mondays (.106), Tuesdays (.114), Wednesdays (.152) and Thursdays (.129). To correct for this potential bias, in all subsequent analyses, Mondays through Saturdays were weighted by their inverse proportions (the weights are 1.57, 1.47, 1.10, 1.29, 0.67 and 0.67 respectively. Despite the considerable variations between these six weights, the descriptive results and the model estimates presented below were almost indistinguishable from the unweighted versions that I used for comparison and robustness checks.

As for most people the start of the day is when they wake up, a day was defined to start at 6am in the morning. Interviewers were suggested to start the interview with the most recent day and proceed backwards in time, but this was not strictly required and other sequences were acceptable if they seemed more convenient for the participant.

Detailed information was collected about the main activities of the adolescents for each hour of the day. Locations of activities were recorded with the help of paper maps printed on A3-size paper. The maps were similar to regular paper maps used for wayfinding and orientation. They were overlaid with a 76×67 grid of numbered 200 by 200m cells. To indicate the location of an activity, participants could point or mention the number of the grid cell (S1 Fig). Over 93% of the time was spent inside the study area, which covered 203km^2^ (of which 182km^2^ are on land). The remaining hours were assigned geographical coordinates with lower resolution, typically the centroid of a town or district in another city. S2 Fig shows where the 843 participants spend their time during the four days recorded in the space-time budget instrument.

Compared to data automatically extracted from mobile phone networks (e.g., [8, 10, 20, 22]), space-time budget data has two disadvantages. First, because of the high fieldwork costs, samples sizes are generally limited to less than 1,000 whereas mobile phone network data typically include over 40,000 phone users. Second, because of participants’ memory limitations, reliable space-time budget data cannot cover more than a few days whereas mobile phone data often cover multiple months or even years. Space-time budget data have three advantages over mobile network data. First, whereas networks only record the locations of phones when they are actively used, space-time budget data include continuous (hour by hour) measures. Second, whereas the spatial resolution of mobile phone network locations is limited by the density of towers, the spatial resolution of space-time budget data can be chosen by the researcher. Third, whereas mobile network data are population data of all phone users whose phone activity level must reach a certain threshold to be useful for research, space-time budget data can be conducted samples that are representative of the population under study.

### Police records

Police records were screened for evidence of crime involvement of the 517 participants who consented that additional information about them be collected. The police records allowed detailed reconstruction of the respondents’ criminal records (i.e., which offenses they had been charged with and the times and locations of these offenses).

Access to police records was provided by the unit The Hague of the Dutch National Police. The police records were drawn from the HKS information system. The HKS system is used for investigative purposes and contains data about all persons who have been suspected of having committed a crime serious enough, and supported by enough evidence, to warrant prosecution. The HKS contains information about the complete criminal record of the individuals. The system has been taken out of service in 2013, making 2012 the last year over which full and reliable crime data can be obtained.

In the police files, records of 70 (14%) of the 517 participants were traced that indicated involvement in criminal conduct. The total number of crime incidents was 178, indicating that some had been involved in multiple crimes. The crimes included a wide variety of property and violent crimes, including theft, burglary, assault, robbery, drug dealing and public order offences. No selections were made with respect to crime type.

Close inspection of the space-time structure of the crime data demonstrates that some pairs of crimes took place on the same day and at the same address. In these cases, it appears that multiple crimes have been reported that were part of the same incident (for example, theft escalating into assault, or drug dealing leading up to robbery). To prevent any conclusions about repeat locations based on artefacts, I selected only the 165 crimes that were the first to take place in a given location on a given day. S1 Table presents the number of offenses per offender. In S3 Fig, I overlaid the locations of the 165 offenses on a map of the aggregated whereabouts of the 70 offenders who committed these offenses.

The crimes took place between at least 35 days and no more than 1484 days (4 years) after the space-time budget interview was administered, with a mean of 791 days (2 years and two months, see S2 Table).

Police data are not collected for the purpose of scientific research, and they are incomplete for two reasons. First, police data on *crime* exclude those crimes that victims did not report to the police. Based on the International Crime Victimization Survey (ICVS), it is estimated that in western countries on average about 50 percent of crime victims report to the police, a figure that is 58 percent in The Netherlands [33]. Second, police data on *offenders* are incomplete because they exclude offenders who are not apprehended. Internationally, the percentage of reported crime for which an offender is identified (the clearance rate) is 34 percent, but it is only 13 percent in the Netherlands [34]. Since both selection mechanisms—reporting and clearance—are unlikely to be random, generalizations based on police data must be made with great caution.

### Points of interest

I used numbers of points of interest as a generic measure of crime opportunity for each of the grid cells in the study area. The points of interest in the period 2008–2010 were extracted from the LISA, a commercially available geo-referenced dataset on all branches of businesses and facilities in The Netherlands, including both commercial and public facilities (government, education, health care, recreation; see www.lisa.nl).

### Radius of gyration

Following prior research in human dynamics (e.g., [10]), the characteristic size of the area covered by the mobility of a single person up to time *t* is indicated by his or her radius of gyration

$$R_{g}(t)=\frac{1}{t}\sqrt{\sum_{i=1}^{t}{\left(\vec{r_{i}}-\vec{r_{c}}\right)}^{2}}$$

where t = 1 … 96 represents the number of hours measured, starting at 6am on the first day, $\vec{r_{i}}$ represents the person’s position at time *i* and $\vec{r_{c}}=\frac{1}{t}\sum_{i}^{t}\left(\vec{r_{i}}\right)$ = is the person’s center of mass. Note that measure is backward-looking only, and thus the center of mass at time *t* is a summary measure of the locations visited up to and including time t. For the complete four days covered in the space-time budget, t = 4 × 24 = 96.

### Predictability

Following [8], the predictability of an individual’s whereabouts was derived from entropy measures of the sequence of 96 locations (i.e., 200 × 200m grid cells) reported in the space-time budget. It varies between 0 (completely random) and 1 (completely determined). *Random predictability* Π^rand^ is a measure of how well an individual’s whereabouts can be foreseen if only the set of unique locations visited is known. *Temporal uncorrelated predictability* Π^unc^ applies to the case in which the number of hours spent at each location is known as well, but not the temporal order of visits, and *maximum predictability* Π^max^ to the case where in addition the temporal order is known too. By definition, Π^max^ > Π^unc^ > Π^rand^.

### Statistical model

I use a discrete spatial choice approach [35] to explain the offender’s choice of a crime location and test the hypotheses. Following the random utility maximization postulate and according to a conditional logit model [36], the offender chooses a single location from the full choice set of 4558 locations (67 × 67 = 5092 grid cells of 200x200m each, minus 544 grid cells completely located on the North Sea). Offender *i* chooses the location *j* that maximizes a utility function

$$U_{ij}=V_{ij}+\varepsilon_{ij}$$ (1)

where *U*_*ij*_ is the utility that offender *i* derives from targeting at location *j*, *V*_*ij*_ is the deterministic part of utility that captures the knowledge of the analyst, and *ε*_*ij*_ is a random disturbance term that captures the analyst’s uncertainty. The deterministic part of utility V_ij_ is a linear function of the attributes of individuals and locations:

$$V_{ij}=\beta_{1}H_{ij}^{A}+\beta_{2}H_{ij}^{B}{+\beta }_{3}H_{ij}^{C}+\beta_{4}E_{ij}^{A}+\beta_{5}E_{ij}^{B}+\beta_{6}E_{ij}^{C}+\beta_{7}E_{ij}^{D}+\beta_{8}E_{ij}^{E}+\beta_{9}C_{ij}^{O}+\beta_{10}C_{ij}^{A}+\beta_{11}C_{ij}^{B}+\beta_{12}C_{ij}^{C}+\beta_{13}C_{ij}^{D}+\beta_{14}C_{ij}^{E}+\beta_{15}F_{j}+\beta_{16}R_{j}+\beta_{17}S_{j}$$ (2)

The seventeen terms on the right-hand side of the equation are a weighted sum of the following attributes of locations and:

$H_{ij}^{A}$ = subject *i* spent 1–4 hours in location *j* (0,1)
$H_{ij}^{B}$ = subject *i* spent 5–16 hours in location *j* (0,1)
$H_{ij}^{C}$ = subject *i* spent 17–96 hours in location *j* (0,1)
$E_{ij}^{A}$ = subject *i* spent time in a location 1^st^ order contiguous to location *j* (0,1)
$E_{ij}^{B}$ = subject *i* spent time in a location 2^nd^ order contiguous to location *j* (0,1)
$E_{ij}^{C}$ = subject *i* spent time in a location 3^rd^ order contiguous to location *j* (0,1)
$E_{ij}^{D}$ = subject *i* spent time in a location 4^th^ order contiguous to location *j* (0,1)
$E_{ij}^{E}$ = subject *i* spent time in a location 5^th^ order contiguous to location *j* (0,1)
$C_{ij}^{O}$ = subject *i* committed a prior crime in location *j* (0,1)
$C_{ij}^{A}$ = subject *i* committed a prior crime in a location 1^st^ order contiguous to location *j* (0,1)
$C_{ij}^{B}$ = subject *i* committed a prior crime in a location 2^nd^ order contiguous to location *j* (0,1)
$C_{ij}^{C}$ = subject *i* committed a prior crime in a location 3^rd^ order contiguous to location *j* (0,1)
$C_{ij}^{D}$ = subject *i* committed a prior crime in a location 4^th^ order contiguous to location *j* (0,1)
$C_{ij}^{E}$ = subject *i* committed a prior crime in a location 5^th^ order contiguous to location *j* (0,1)
$F_{j}^{A}$ = at least one catering business in location *j* (0,1)
$R_{j}^{B}$ = at least one retail business in location *j* (0,1)
$S_{j}^{C}$ = at least one school in location *j* (0,1)

S4 Fig provides a visual example of how variables were coded. I calculated spatially contiguous bands up to the 6^th^ order. Because I did not find significant effects beyond 5^th^ order contiguity, and because the literature suggests proximity effects up to 2 or 4 bands (200–400 meter), I did not calculate contiguity beyond the 6^th^ order.

The values of β_1_ to β_17_ are parameters that are estimated from the data. Their sizes, relative to 0 and relative to each other, their directions and their standard errors determine whether the hypotheses are confirmed or rejected (according to the theory, all β > 0). The values *e*^*β*^ are odds ratios, and represent the factor by which the odds of being chosen increase (odds ratio > 1) or decrease (odds ratio < 1) with each one-unit increase of the right-hand side variables. As all right-hand side variables are binary, the odds ratios are ratios of the odds of the ‘1’ category and the odds of the ‘0’ category.

If the unobserved random utility components *ε*_*ij*_ are independently and identically distributed according to an extreme value distribution, the conditional logit model [36] can be derived. This statistical model allows us to estimate the β parameters from empirical data. According to the conditional logit model, the probability that offender *i* chooses location *j* is given by:

$$P_{ij}=\frac{e^{V_{ij}}}{\sum_{k=1}^{4558}e^{V_{ik}}}$$ (3)

### Evaluation of predictions

To evaluate the quality of the prediction, I use a simple and intuitive measure. After model estimation, for each crime in the data all 4558 locations in the study area are lined up in ascending order of predicted probability P_ij_ according to the model estimates. The model fit measure is the rank number of the grid cell where the crime was actually committed, divided by the total number of 4558 grid cells, averaged over all crimes. It reflects the proportion of the study area with a lower average predicted probability than the locations actually chosen. The value would normally range between 1 (perfect prediction) and .50 (the expected average value for a null model with random predictions) although predictions worse than random are possible. Average rank orders are assigned in case of ties (i.e. multiple grid cells with equal predicted probabilities).

## Results

### Mobility differences between sample subgroups

Before testing hypotheses, I describe four key features of the mobility of the study participants, argue that the observed patterns are consistent with those reported in prior research with different samples and methods [8, 10, 20], and demonstrate that the patterns of (future) offenders are hardly different from those of others.

For all groups, the number of unique locations roughly follows a Gaussian distribution in the range 1–15, with a median value of 6 locations. Individuals spend 60 percent of the time in their most frequently visited location (home), and another 15 percent in their second most visited location (typically school), their radius of gyration follows a lognormal distribution with a mean of 3.2 km, and their predictability Π^max^ equals 0.96 on average, slightly above the values found in general populations by prior research [8, 19].

**Fig 1:**
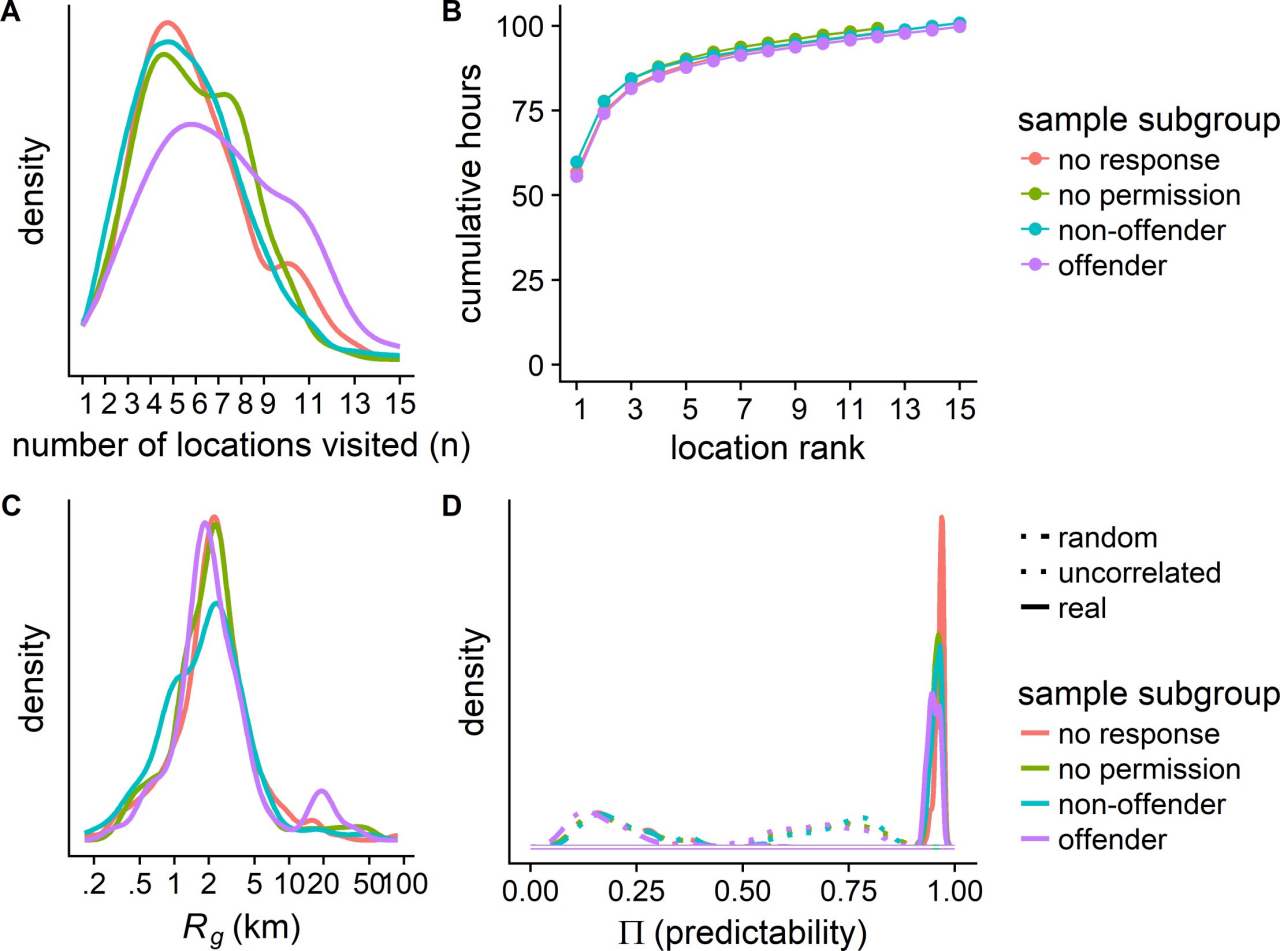
Key features of mobility are similar amongst subgroups of the sample. The sample can be segmented into four mutually exclusive subgroups: (1) participants (n = 228) who could not be asked for permission to link their registry data to their survey responses, (2) participants (n = 98) who did not give permission to link their registry data to their survey responses, (3) participants (n = 447) who gave permission and did not commit crimes subsequently, and (4) participants (n = 70) who gave permission and who became offenders during the study period. (**A**) On average, offenders visited 1 location more than individuals in the other three groups (see S4 Table). (**B**) All participants spent about 75 percent of their time in their two most visited locations (home and school). (**C**) The radius of gyration is approximately 3km and displays minor but insignificant differences between the four groups (see S5 Table). (**D**) The three predictability measures differed slightly across the groups, with a very slight tendency for offenders to be less predictable (see S6–S8 Tables). Densities in (A), (C) and (D) were calculated with the Gaussian kernel density estimator proposed by Scott [37]. Values are not labeled along the Y axis is they have no useful interpretation here.

As the four panels in Fig 1 also show, offenders are very similar both to non-offenders and to the two groups of whom it is unknown whether they are offenders. Except for the finding that on average the unique number of visited locations is somewhat larger for offenders than for the other groups, the cumulative numbers of locations visited, the radius of gyration and the predictability measures are similar across the four groups. Thus, as far as routine mobility is concerned, adolescent offenders are not very different from other adolescents.

The key features themselves are similar to those reported elsewhere on different samples and with different spatial and temporal scales [8, 10, 20], although the short observation period of four days and the fixed temporal resolution of one-hour slots are measurement limitations that likely underestimate the total number of visited locations observed [22] but not the radius of gyration. Furthermore, the young age of the sampled cohorts most likely causes the mean radius of gyration to be smaller than in the general population, because the average commute to school is shorter than the average commute to work.

**Fig 2:**
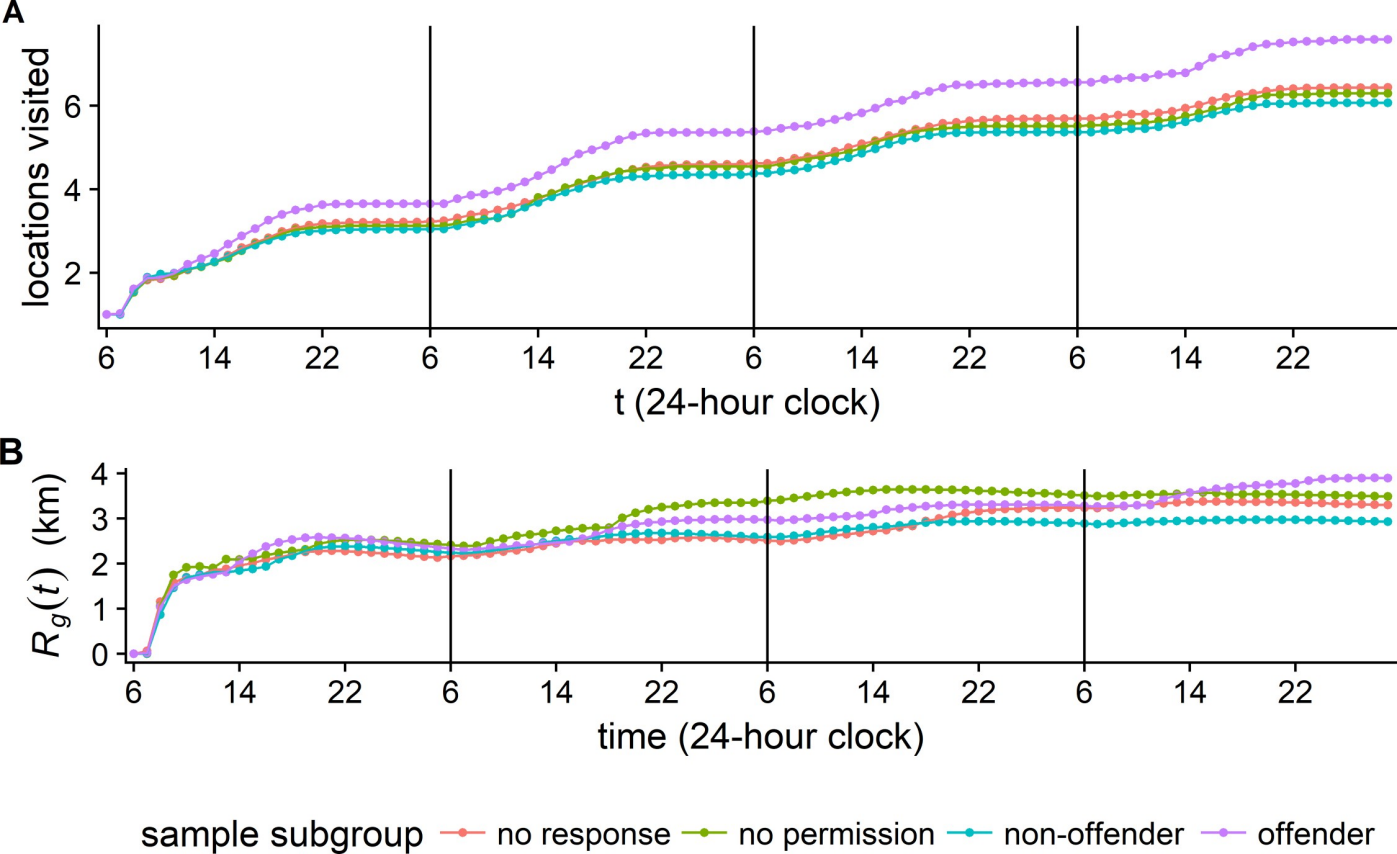
Development of number of unique locations visited and radius of gyration over time. (A) The cumulative number of visited locations grows linearly over time, remains stable during sleeping hours and does not reach an asymptotic stable level within the observation period of four days. (B) Apart from the first few hours on the first day covered, the radius of gyration grows sub-linearly, slightly decreases during sleep due to 9 hours of immobility, and reaches a stable level of approximately 3km after two days already. The adolescents slept almost 9 hours per day on average, typically at home during the night between 22:00 and 7:00.

Fig 2 demonstrates how the cumulative number of locations visited and the radius of gyration develop over the four days reported in the space-time budget interviews.

### Modeling location choice and hypothesis tests

Within four years after completing the first space-time budget interview, 70 (14%) of the 517 who had given permission to have their data linked to other sources had committed at least one offense in the study catchment area that was brought to the attention of the police. Together these 70 participants committed 165 detected offenses in the study area. Based on their whereabouts reported in the space-time budget interview and on previously committed offences registered by the police, I attempt to predict the locations of subsequent crimes that the offenders perpetrated. I use a discrete spatial choice approach to explain the offender’s choice of a crime location and test the hypotheses.

**Fig 3:**
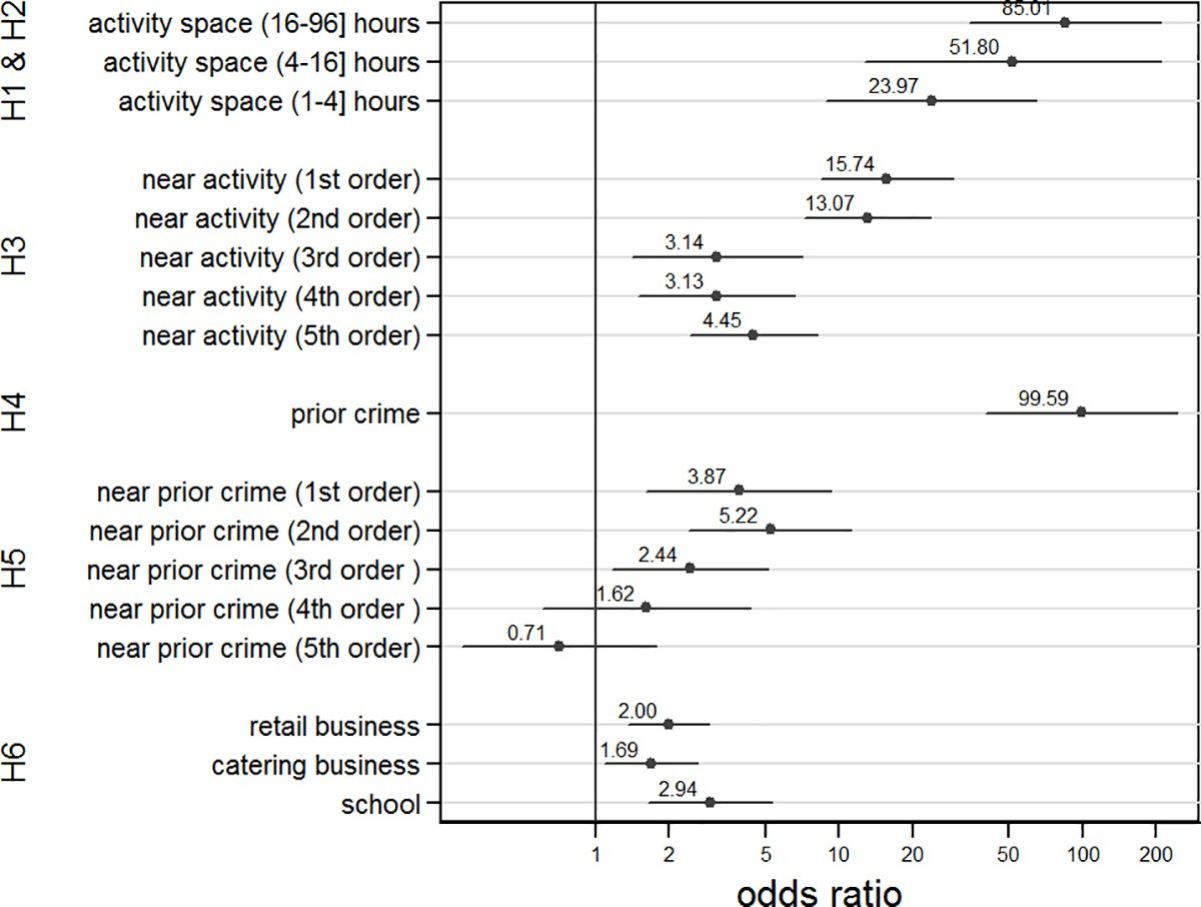
Quality of the explanation and tests of the hypotheses. Estimated effects (dots) and 95% confidence intervals (lines) of the conditional logit model (70 adolescents, 165 crimes and 4558 locations). **H1** (activity space): All locations within activity space (activity 1–96 hours) are more likely to be chosen for committing crime than those outside activity space. **H2** (usage): Frequently used locations are more likely to be targeted than locations less often used (non-significant). **H3** (exploration): The likelihood of being targeted decreases with the distance from activity space. **H4** (repeat location): previously targeted locations are more likely to be targeted than locations not previously targeted. **H5** (near repeat location). The likelihood of being selected decreases with the distance from the previous crime (partially and non-significant). **H6** (opportunity) Locations with schools, retail businesses and catering businesses are more likely targets.

The findings reported in Fig 3 provide support for most of the six hypotheses. Except for the presence of retail and catering businesses, estimates and confidence intervals of all attributes are far above the baseline value of 1, and are thus statistically significant. Therefore, locations in and around offenders’ activity spaces and prior crime locations are much more likely to be targeted than other locations. The estimated differences on the temporal and spatial dimensions—hours spent at a location, and distance from a location—do confirm the expected pattern, but they are not all statistically significant. For example, grid cells that are first-order contiguous to the offender’s activity space (*near activity (1*^*st*^*)*) are not significantly more likely chosen than those that are second-order contiguous, although both are more likely chosen than those that are third-order contiguous. Similarly, differences between first-order, second-order and third-order contiguity to prior crime locations are not statistically significant. More generally, the confidence intervals around the point estimates are rather large. This is partly due to the sparseness of the data (165 crimes relative to the total number of 4558 grid cells), but it also indicates that there is a fair amount of heterogeneity that is not covered by the explanatory variables.

### Accuracy of predictions

**Fig 4:**
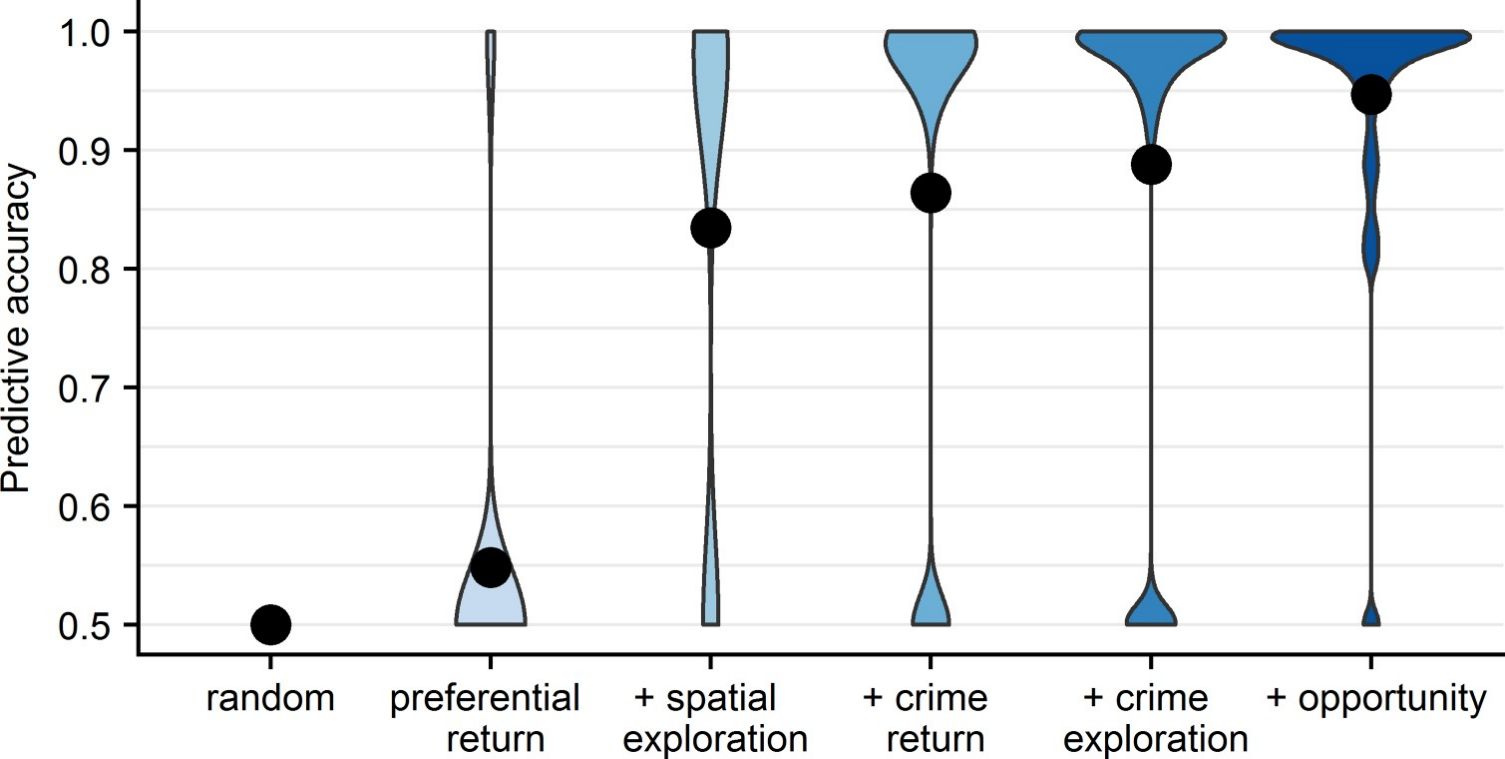
Accuracy of the predictions. A simple and intuitive measure of model fit is the proportion P of potential crime locations with a lower predicted probability than the actual crime location. P = 1 in case of a model with perfect prediction, while P = .5 in case of random choice. Increasing model complexity along the horizontal axis improves predictive quality (black dots). The shape of the density (blue ‘hourglasses’) of the accuracy measure P show thats its distribution is bimodal, and that model fit improvement is a function of individuals ‘jumping’ from the ‘almost random’ category to the ‘near-perfect’ category when variables are added to a model. The accuracy measure P correlates .96 with McFadden’s R2, a common but less interpretable measure of model fit. In S15 Table I present the results of a parsimonious model that uses continuous measures of time use and proximity. This model cannot test all hypotheses, but its predictions are almost equally accurate (P = .94) as those of the most comprehensive model presented here (“+ opportunities”, P = .95).

For six models of increasing complexity (see S10–S14 Tables), the predictive accuracy is displayed in Fig 4. Taking into account that the individuals’ activity spaces were measured during only four days and on average 21 months before their first crime during the observation period, the predictive accuracy is surprisingly high, with the true location situated in the 95^th^ percentile of the predicted locations in the most comprehensive model. Without the inclusion of opportunity structure measures (which are not included in the preferential return and spatial exploration model), the true location is in the 83^rd^ percentile, still high but substantially lower, and probably reflecting that four days is a period too short to fully capture our complete activity spaces.

## Discussion

The findings reported here suggest that all of us, law-breakers and law-abiders alike, may be less innovative and more predictable than we think we are, or would like to be. Although crime may be an exceptional activity, its location can be predicted by preferential return and spatial exploration, the same two mechanisms that generate our routine mobility. In line with the findings from prior studies, but based on direct measures of individual activity spaces, I demonstrated that offenders not only tend to commit crimes in and around the places they have visited before when pursuing either their legal daily activities, but also have a tendency to commit crimes in and around the locations of their previous crimes. Although low crime reporting rates and low clearances rates require cautious conclusions, the findings do seem to support the general assumption of environmental criminology that legal and illegal conduct are not fundamentally different, and follow the same behavioral laws.

The findings demonstrate that in addition to activity space, criminal opportunity is relevant in its own right for explaining and predicting where crimes take place, even if we condition on offenders' activity spaces and prior crime locations. This may partly signal two limitations of the activity space measure: it covers only four days and was administered only at a single moment in time. Concentrations of businesses and facilities are probably part of many adolescents’ activity space—or are likely to become part of it as they grow older and become more mobile—even if they are not frequently visited. Furthermore, the measurement of the participants’ whereabouts took place October-May, a period during which the maximum daily temperature averages ~10° Celsius. It did not include the summer (June-September, when maximum daily temperature averages ~20° Celsius) a time of year during which young people in the age group of the sample spend less time in home-based sedentary activities and more time outdoors [38, 39]. Outdoor activities away from home probably provide more opportunities for offending than indoor activities. Nevertheless, the role of opportunity in crime location choices also highlights that some crimes may actually be committed at criminogenic places located outside and distant from the offender's activity space. For example, co-offenders may completely rely on the spatial knowledge of their accomplices.

The findings presented here will not directly help prevent crime, because they do not inform us about who the prospective offenders are and when they will commit crimes. Without that knowledge, the use of existing epidemiological and seismological methods for crime prediction is likely more efficient, as their predictions are based on data (location, time and crime type) that are already collected routinely for law enforcement purposes. However, armed with knowledge of who are most likely to commit crimes—crime is dominated by adolescents and by people with a history of prior offending—the findings can help the police and other law enforcement agencies to direct interventions to places frequently visited by potential motivated offenders.

The reported findings can be particularly useful in criminal investigations, when crimes have taken place and the police searches for the identity and whereabouts of the offenders using geographic profiling, a set of principles and methods to prioritize the most likely offender of a single crime or a series of crimes based on the locations and timings of these crimes [40]. Given what we have learned here about preferential return and spatial exploration in criminal behavior, criminal investigations should generally prioritize suspects who are familiar with the location of the crime and its environs, either because of their legal routine activities or because of their prior offences.

## Supporting information

S1 Fig The 76 × 67 grid of the study area. Orientation rotated for compatibility with common paper maps of The Hague. Grid cells in North Sea removed. The inset shows a detail of the map presented to participants.
(TIF)

S2 Fig Map of time use by all participants. Overview of where the 843 participants spent over 6,700 hours during four days in each of the 4558 grid cells on land. Schools stand out as frequently visited locations, as they were places of convergence for the participants during the three weekdays recorded.
(TIFF)

S3 Fig Map of time use and offenses by offenders. Overview of where the 70 offenders spent over 81,000 hours during four days in each of the 4558 grid cells on land. Overlaid (in red) are the locations of the offenses they committed during four years after the time use space-time budget measurement.
(TIFF)

S4 Fig Example of the coding of variables. For a single random individual in the dataset, the maps shows how much time s/he spent in each 200x200m grid cell in the study area during the recorded four days (4x24 hours). The red grid cells constitute the individual’s activity space. The number of hours spent per grid cell is indicated by three shades of red. The map also displays how six contiguity bands were constructed individual’s activity space. To protect the privacy of the individual, the activity space and contiguity bands on the map have been randomly moved between -5 and 5 positions horizontally and vertically along the grid axes, i.e. maximally 1 kilometer in all directions.
(TIFF)

S1 Table Total number of offenses per offender (N = 165 offenses). (DOCX)

S2 Table Period between space-time budget interview and offense (N = 165 offenses). (DOCX)

S3 Table Proximity of offense locations to offenders’ activity spaces (N = 165 offenses). (DOCX)

S4 Table Means, medians and statistical significance of Wilcoxon rank-sum tests of the number of unique locations visited by (1) non-responders, (2) non-compliers (3) non-offenders (4) offenders. (DOCX)

S5 Table Means, medians and statistical significance of Wilcoxon rank-sum tests of the radius of gyration of (1) non-responders, (2) non-compliers (3) non-offenders (4) offenders. (DOCX)

S6 Table Means, medians and statistical significance of Wilcoxon rank-sum tests of the random predictability of (1) non-responders, (2) non-compliers (3) non-offenders (4) offenders. (DOCX)

S7 Table Means, medians and statistical significance of Wilcoxon rank-sum tests of the uncorrelated predictability of (1) non-responders, (2) non-compliers (3) non-offenders (4) offenders. (DOCX)

S8 Table Means, medians and statistical significance of Wilcoxon rank-sum tests of the real predictability of (1) non-responders, (2) non-compliers (3) non-offenders (4) offenders. (DOCX)

S9 Table Descriptive statistics of variables used in the conditional logit models (Fig 3, Fig 4, S14 Table). The total N is 165 (crimes) × 4558 (grid cells) = 752,070. All 17 variables are binary and have a minimum value of 0 and a maximum value of 1.
(DOCX)

S10 Table Conditional logit estimates of model “Preferential return” (Fig 4). Descriptive statistics of the covariates are presented in S9 Table.
(DOCX)

S11 Table Conditional logit estimates of model “+ spatial exploration” (Fig 4). Descriptive statistics of the covariates are presented in S9 Table.
(DOCX)

S12 Table Conditional logit estimates of model “+ prior crime” (Fig 4). Descriptive statistics of the covariates are presented in S9 Table.
(DOCX)

S13 Table Conditional logit estimates of model “+ prior crime exploration” (Fig 4). Descriptive statistics of the covariates are presented in S9 Table.
(DOCX)

S14 Table Conditional logit estimates of model “+ opportunity” (Fig 3 and Fig 4). Descriptive statistics of the covariates are presented in S9 Table.
(DOCX)

S15 Table Conditional logit estimates of a parsimonious version of the model “+ opportunity”. Distance from activity space and distance from prior crime are measured as the contiguity order of a grid cell, with a maximum of 7 for any grid cells beyond 6th order contiguity, and 0 for grid cells inside activity space and grid cells were the offender committed a prior offense, respectively.
(DOCX)
