# Supplementary figures and images for: Adolescent offenders' current whereabouts predict locations of their future crimes
Source: PLoS One. 2019 Jan 30;14(1):e0210733. doi: 10.1371/journal.pone.0210733 (PMC6353130; doi:10.1371/journal.pone.0210733)

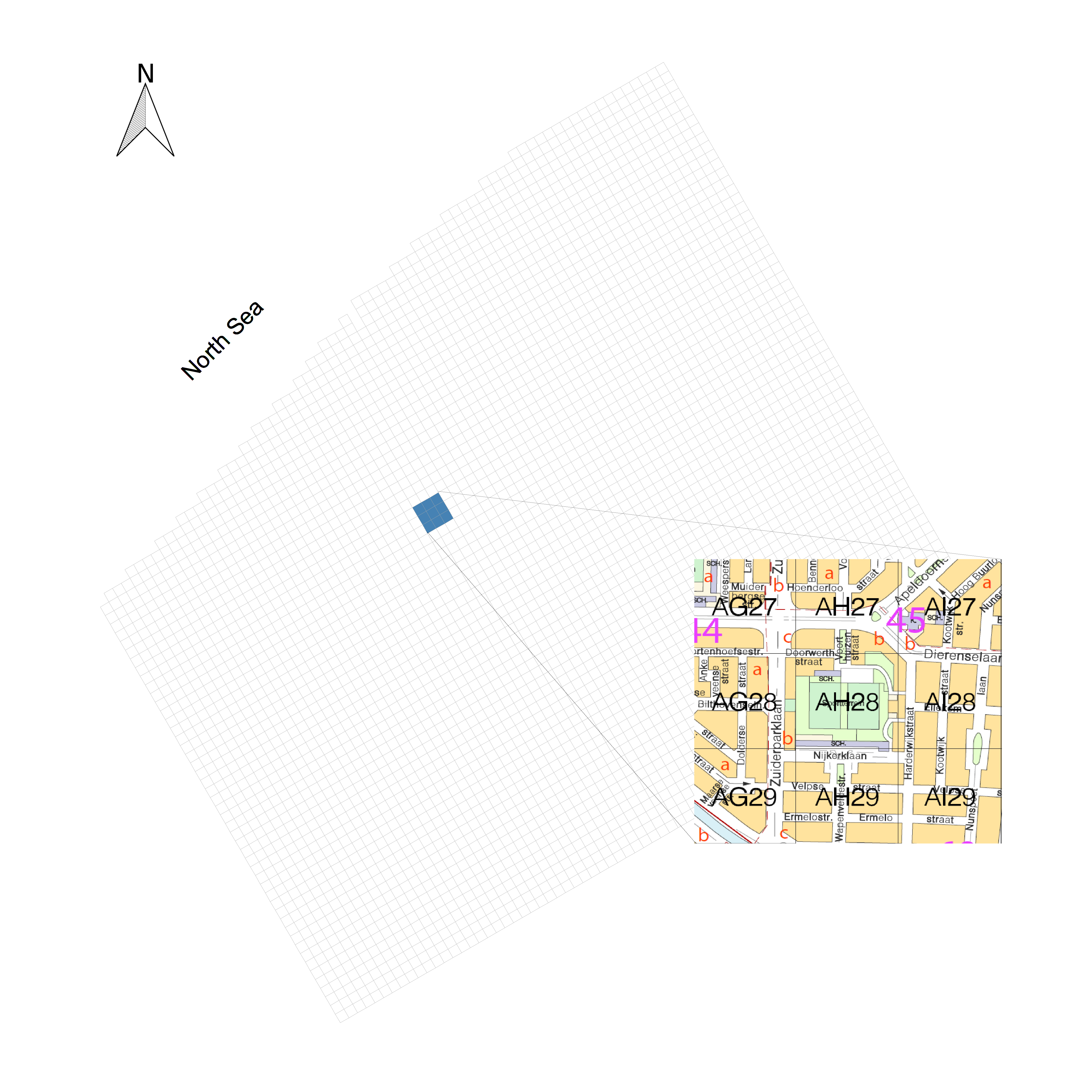

Supplement: S1 Fig — Orientation rotated for compatibility with common paper maps of The Hague. Grid cells in North Sea removed. The inset shows a detail of the map presented to participants. (TIF) [file pone.0210733.s001.tif]

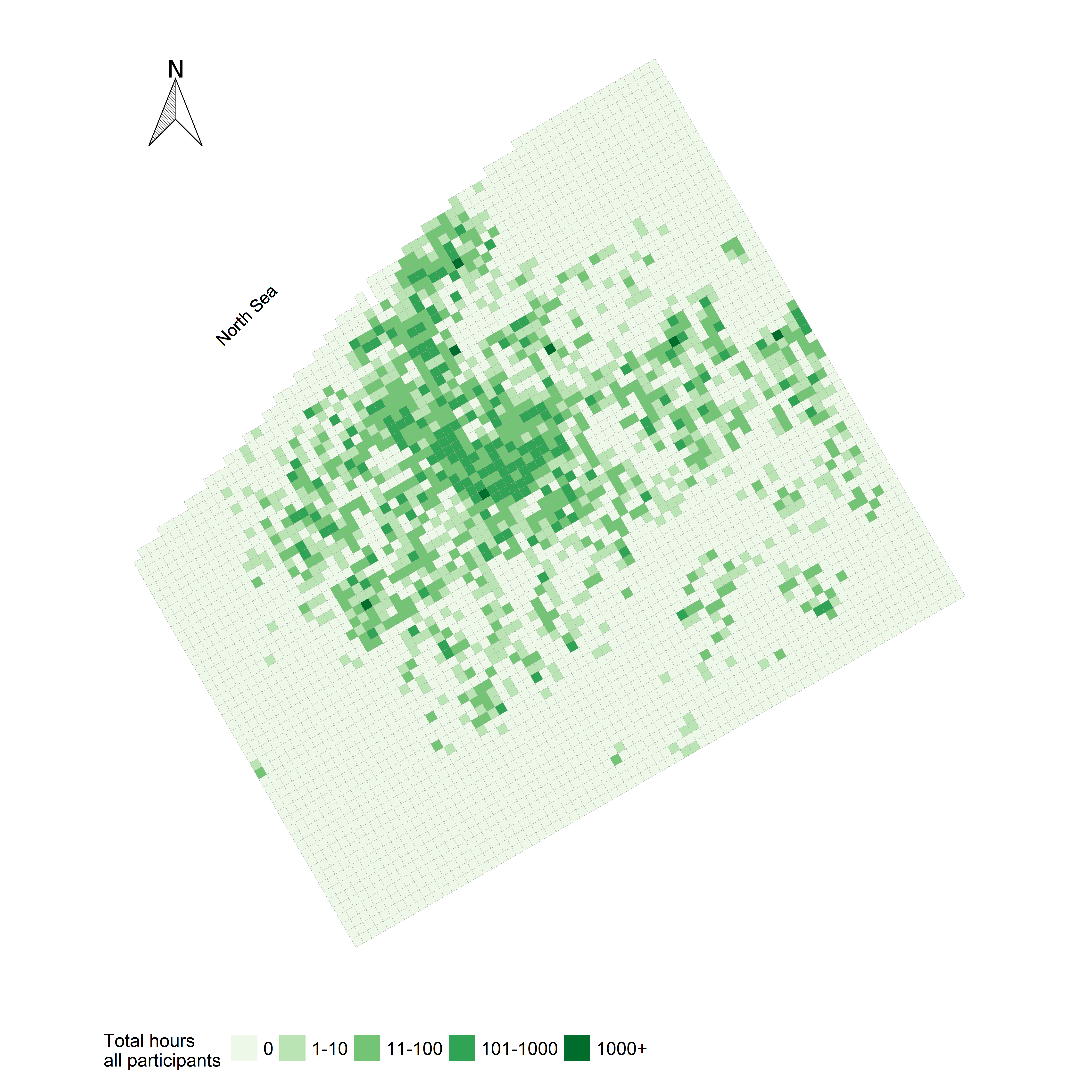

Supplement: S2 Fig — Overview of where the 843 participants spent over 6,700 hours during four days in each of the 4558 grid cells on land. Schools stand out as frequently visited locations, as they were places of convergence for the participants during the three weekdays recorded. (TIFF) [file pone.0210733.s002.tiff]

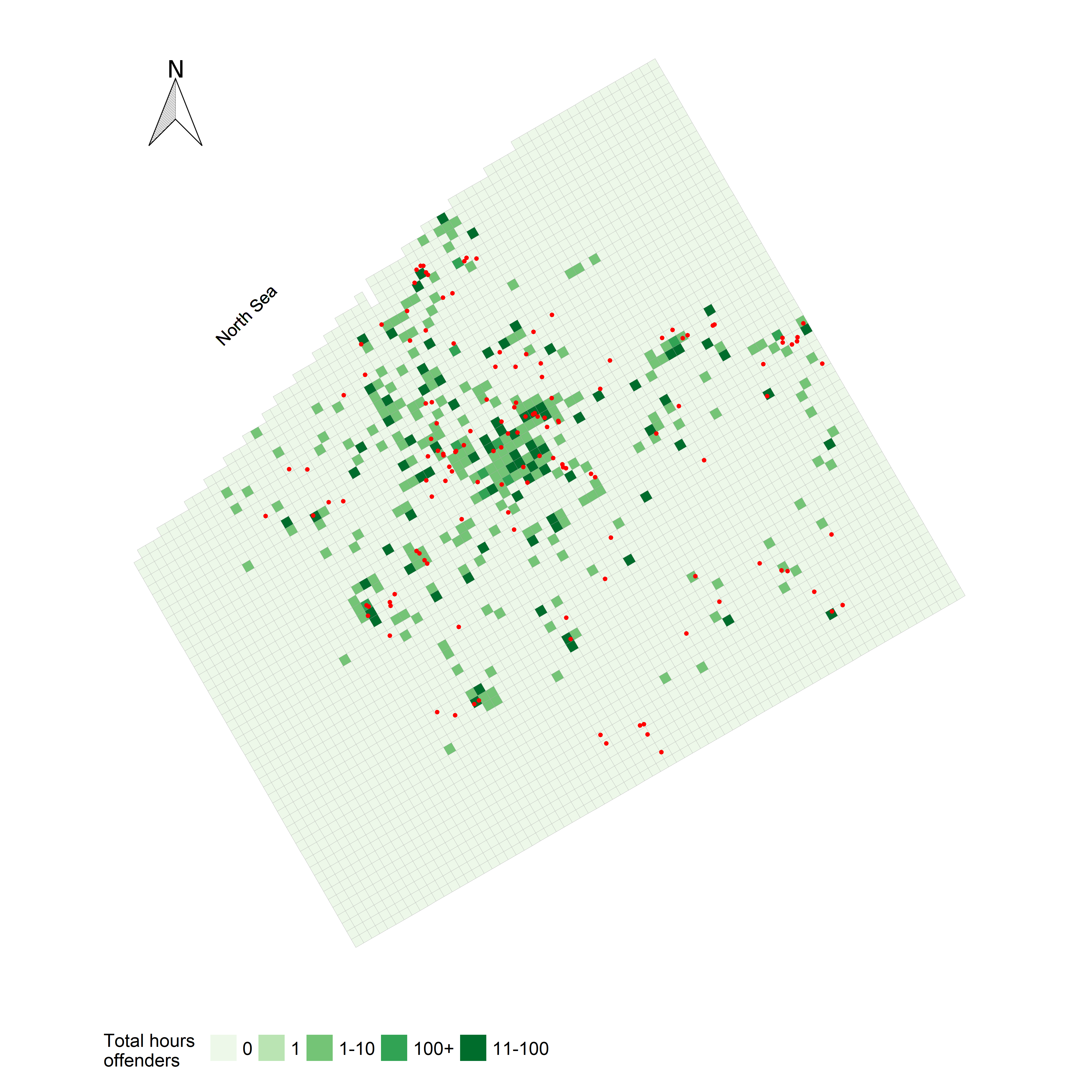

Supplement: S3 Fig — Overview of where the 70 offenders spent over 81,000 hours during four days in each of the 4558 grid cells on land. Overlaid (in red) are the locations of the offenses they committed during four years after the time use space-time budget measurement. (TIFF) [file pone.0210733.s003.tiff]

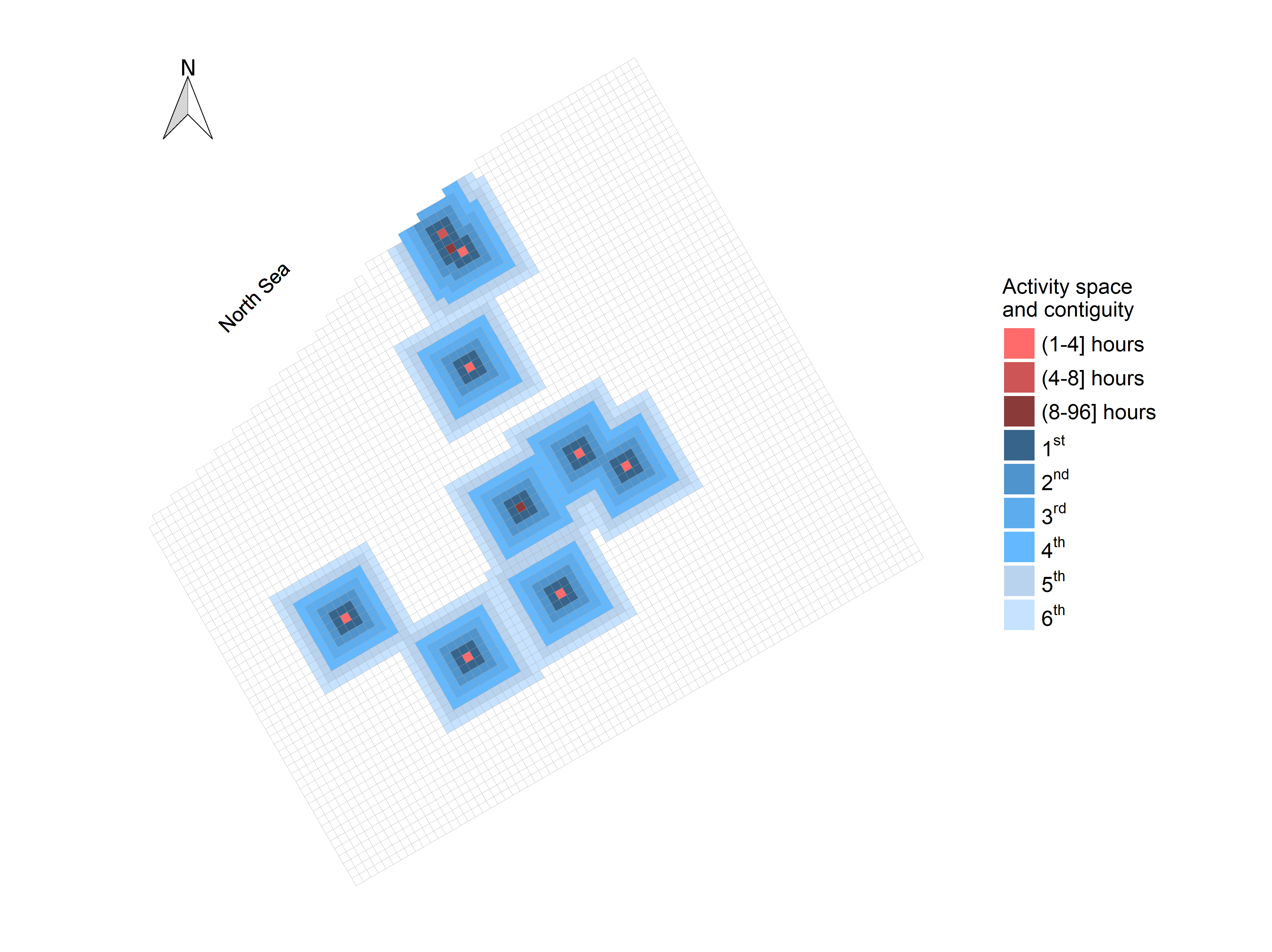

Supplement: S4 Fig — For a single random individual in the dataset, the maps shows how much time s/he spent in each 200x200m grid cell in the study area during the recorded four days (4x24 hours). The red grid cells constitute the individual’s activity space. The number of hours spent per grid cell is indicated by three shades of red. The map also displays how six contiguity bands were constructed individual’s activity space. To protect the privacy of the individual, the activity space and contiguity bands on the map have been randomly moved between -5 and 5 positions horizontally and vertically along the grid axes, i.e. maximally 1 kilometer in all directions. (TIFF) [file pone.0210733.s004.tiff]
