# Supplementary material for: Adolescent offenders' current whereabouts predict locations of their future crimes
Source: PLoS One. 2019 Jan 30;14(1):e0210733. doi: 10.1371/journal.pone.0210733 (PMC6353130; doi:10.1371/journal.pone.0210733)
Supplement: S1 Table — (DOCX) [file pone.0210733.s005.docx]

S1 Table. Total number of offenses per offender (N=165 offenses)

| Offenses | # | % |
| --- | --- | --- |
| 1 offense | 35 | 50.0 |
| 2 offenses | 15 | 21.4 |
| 3 offenses | 11 | 15.7 |
| 5 offenses | 3 | 4.3 |
| 7 offenses | 1 | 1.4 |
| 8 offenses | 2 | 2.9 |
| 9 offenses | 1 | 1.4 |
| 10 offenses | 2 | 2.9 |
| Total | 70 | 100 |
