# Supplementary material for: Adolescent offenders' current whereabouts predict locations of their future crimes
Source: PLoS One. 2019 Jan 30;14(1):e0210733. doi: 10.1371/journal.pone.0210733 (PMC6353130; doi:10.1371/journal.pone.0210733)
Supplement: S2 Table — (DOCX) [file pone.0210733.s006.docx]

S2 Table. Period Between Space-Time Budget Interview and Offense (N=165 offenses)

| Period | # | % |
| --- | --- | --- |
| 0-1 year | 30 | 18.2 |
| 1-2 years | 39 | 23.6 |
| 2-3 years | 55 | 33.3 |
| 3-4 years | 41 | 24.9 |
| Total | 165 | 100 |
