# Supplementary material for: Adolescent offenders' current whereabouts predict locations of their future crimes
Source: PLoS One. 2019 Jan 30;14(1):e0210733. doi: 10.1371/journal.pone.0210733 (PMC6353130; doi:10.1371/journal.pone.0210733)
Supplement: S3 Table — (DOCX) [file pone.0210733.s007.docx]

S3 Table. Proximity of Offense Locations to Offenders’ Activity Spaces (N=165 offenses)

| Proximity to activity space | # grid cells | # offenses | λ^1^ |
| --- | --- | --- | --- |
| Activity space (0-200m) (16-96] hours | 263 | 9 | 3.42 |
| Activity space (0-200m) (4-16] hours | 230 | 2 | 0.87 |
| Activity space (0-200m) (1-4] hours | 737 | 5 | 0.68 |
| (Total) Activity space (0-200m) | 1,230 | 16 | 1.30 |
| 1st order contiguity (200-400m) | 7,992 | 33 | 0.41 |
| 2nd order contiguity (400-600m) | 13,024 | 30 | 0.23 |
| 3rd order contiguity (600-800m) | 16,408 | 7 | 0.04 |
| 4rd order contiguity (800-1000m) | 18,967 | 8 | 0.04 |
| 5th order contiguity (1000-1200m) | 20,885 | 12 | 0.06 |
| 6th order and beyond | 673,564 | 59 | 0.01 |
| Total | 752,070 | 165 | 100 |

^1^ λ = 100 × number of offenses / number of grid cells, i.e. 100 times the probability that a grid cell is the location of an offense given its proximity to the offender’s activity space.
