# Supplementary material for: Adolescent offenders' current whereabouts predict locations of their future crimes
Source: PLoS One. 2019 Jan 30;14(1):e0210733. doi: 10.1371/journal.pone.0210733 (PMC6353130; doi:10.1371/journal.pone.0210733)
Supplement: S5 Table — (DOCX) [file pone.0210733.s009.docx]

S5 Table. Means, medians and statistical significance of Wilcoxon rank-sum (Mann-Whitney U) tests of the radius of gyration of (1) non-responders, (2) non-compliers (3) non-offenders (4) offenders.

|  | Offender | Non-offender | Non-responder | Non-complier |
| --- | --- | --- | --- | --- |
| Offender |  |  |  |  |
| Non-offender | n.s. |  |  |  |
| Non-responder | n.s. | n.s. |  |  |
| Non-complier | n.s. | n.s. | n.s. |  |
|  |  |  |  |  |
| Mean | 3.90 | 2.93 | 3.30 | 3.49 |
| Median | 1.91 | 2.00 | 2.21 | 2.14 |
| N | 70 | 447 | 228 | 98 |

* = p < .01, n.s. = p > .01
