# Supplementary material for: Adolescent offenders' current whereabouts predict locations of their future crimes
Source: PLoS One. 2019 Jan 30;14(1):e0210733. doi: 10.1371/journal.pone.0210733 (PMC6353130; doi:10.1371/journal.pone.0210733)
Supplement: S6 Table — (DOCX) [file pone.0210733.s010.docx]

S6 Table. Means, medians and statistical significance of Wilcoxon rank-sum (Mann-Whitney U) tests of the **random** predictability of (1) non-responders, (2) non-compliers (3) non-offenders (4) offenders.

|  | Offender | Non-offender | Non-responder | Non-complier |
| --- | --- | --- | --- | --- |
| Offender |  |  |  |  |
| Non-offender | * |  |  |  |
| Non-responder | * | n.s. |  |  |
| Non-complier | * | n.s. | n.s. |  |
|  |  |  |  |  |
| Mean | .179 | .220 | .207 | .209 |
| Median | .162 | .188 | .188 | .188 |
| N | 70 | 447 | 228 | 98 |

* = p < .01, n.s. = p > .01
