# Supplementary material for: Adolescent offenders' current whereabouts predict locations of their future crimes
Source: PLoS One. 2019 Jan 30;14(1):e0210733. doi: 10.1371/journal.pone.0210733 (PMC6353130; doi:10.1371/journal.pone.0210733)
Supplement: S8 Table — (DOCX) [file pone.0210733.s012.docx]

S8 Table. Means, medians and statistical significance of Wilcoxon rank-sum (Mann-Whitney U) tests of the **real** predictability of (1) non-responders, (2) non-compliers (3) non-offenders (4) offenders.

|  | Offender | Non-offender | Non-responder | Non-complier |
| --- | --- | --- | --- | --- |
| Offender |  |  |  |  |
| Non-offender | * |  |  |  |
| Non-responder | * | * |  |  |
| Non-complier | n.s. | n.s. | * |  |
|  |  |  |  |  |
| Mean | .950 | .956 | .964 | .955 |
| Median | .950 | .957 | .966 | .955 |
| N | 70 | 447 | 228 | 98 |

* = p < .01, n.s. = p > .01
