# Supplementary material for: Adolescent offenders' current whereabouts predict locations of their future crimes
Source: PLoS One. 2019 Jan 30;14(1):e0210733. doi: 10.1371/journal.pone.0210733 (PMC6353130; doi:10.1371/journal.pone.0210733)
Supplement: S9 Table — The total N is 165 (crimes) × 4558 (grid cells) = 752,070. All 17 variables are binary and have a minimum value of 0 and a maximum value of 1. (DOCX) [file pone.0210733.s013.docx]

S9 Table. Means and standard deviations of variables used in the conditional logit models (Fig. 3, Fig. 4, S14 Table). Total N = 165 (crimes) × 4558 (grid cells) = 752,070. All 17 variables are binary and have a minimum value of 0 and a maximum value of 1.

| Variable | Mean | S.D. |
| --- | --- | --- |
| Activity space (16-96] hours | .0004 | .0201 |
| Activity space (4-16] hours | .0003 | .0174 |
| Activity space (1-4] hours | .0009 | .0304 |
| Near activity (1^st^ order) | .0106 | .1024 |
| Near activity (2^nd^ order) | .0173 | .1303 |
| Near activity (3^rd^ order) | .0218 | .1459 |
| Near activity (4^th^ order) | .0252 | .1566 |
| Near activity (5^th^ order) | .0277 | .1641 |
| Prior crime | .0004 | .0192 |
| Near prior crime (1^st^ order) | .0027 | .0519 |
| Near prior crime (2^nd^ order) | .0052 | .0720 |
| Near prior crime (3^rd^ order) | .0077 | .0875 |
| Near prior crime (4^th^ order) | .0102 | .1005 |
| Near prior crime (5^th^ order) | .0124 | .1106 |
| Retail business | .2813 | .4496 |
| Catering business | .1832 | .3868 |
| School | .0259 | .1588 |
