# Supplementary material for: Adolescent offenders' current whereabouts predict locations of their future crimes
Source: PLoS One. 2019 Jan 30;14(1):e0210733. doi: 10.1371/journal.pone.0210733 (PMC6353130; doi:10.1371/journal.pone.0210733)
Supplement: S10 Table — Descriptive statistics of the covariates are presented in S9 Table. (DOCX) [file pone.0210733.s014.docx]

S10 Table. Conditional logit estimates of model “Preferential return” (Figure 4). Descriptive statistics of the covariates are presented in S9 Table.

| Variable | OR | 95% C.I. | p |
| --- | --- | --- | --- |
| Activity space (16–96] hours | 149.58 | 57.43–389.65 | < .001 |
| Activity space (4–16] hours | 44.09 | 10.15–191.59 | < .001 |
| Activity space (1–4] hours | 36.49 | 10.62–125.39 | < .001 |
| Near activity (1^st^ order) |  |  |  |
| Near activity (2^nd^ order) |  |  |  |
| Near activity (3^rd^ order) |  |  |  |
| Near activity (4^th^ order) |  |  |  |
| Near activity (5^th^ order) |  |  |  |
| Prior crime |  |  |  |
| Near prior crime (1^st^ order) |  |  |  |
| Near prior crime (2^nd^ order) |  |  |  |
| Near prior crime (3^rd^ order) |  |  |  |
| Near prior crime (4^th^ order) |  |  |  |
| Near prior crime (5^th^ order) |  |  |  |
| Retail business |  |  |  |
| Catering business |  |  |  |
| School |  |  |  |
| Crimes | 165 |  |  |
| Locations | 4558 |  |  |
| Accuracy | .55 |  |  |
| McFadden Pseudo R^2^ | .04 |  |  |
