# Supplementary material for: Adolescent offenders' current whereabouts predict locations of their future crimes
Source: PLoS One. 2019 Jan 30;14(1):e0210733. doi: 10.1371/journal.pone.0210733 (PMC6353130; doi:10.1371/journal.pone.0210733)
Supplement: S11 Table — Descriptive statistics of the covariates are presented in S9 Table. (DOCX) [file pone.0210733.s015.docx]

S11 Table. Conditional logit estimates of model “+ spatial exploration” (Figure 4). Descriptive statistics of the covariates are presented in S9 Table.

| Variable | OR | 95% C.I. | p |
| --- | --- | --- | --- |
| Activity space (16–96] hours | 340.50 | 123.89–935.81 | < .001 |
| Activity space (4–16] hours | 107.79 | 24.01–483.97 | < .001 |
| Activity space (1–4] hours | 87.88 | 24.50–315.22 | < .001 |
| Near activity (1^st^ order) | 50.09 | 32.61–76.93 | < .001 |
| Near activity (2^nd^ order) | 27.84 | 15.75–49.22 | < .001 |
| Near activity (3^rd^ order) | 5.13 | 2.36–11.15 | < .001 |
| Near activity (4^th^ order) | 5.06 | 2.24–11.42 | < .001 |
| Near activity (5^th^ order) | 6.87 | 3.55–13.30 | < .001 |
| Prior crime |  |  |  |
| Near prior crime (1^st^ order) |  |  |  |
| Near prior crime (2^nd^ order) |  |  |  |
| Near prior crime (3^rd^ order) |  |  |  |
| Near prior crime (4^th^ order) |  |  |  |
| Near prior crime (5^th^ order) |  |  |  |
| Retail business |  |  |  |
| Catering business |  |  |  |
| School |  |  |  |
| Crimes | 165 |  |  |
| Locations | 4558 |  |  |
| Accuracy | .81 |  |  |
| McFadden Pseudo R^2^ | .15 |  |  |
