# Supplementary material for: Adolescent offenders' current whereabouts predict locations of their future crimes
Source: PLoS One. 2019 Jan 30;14(1):e0210733. doi: 10.1371/journal.pone.0210733 (PMC6353130; doi:10.1371/journal.pone.0210733)
Supplement: S12 Table — Descriptive statistics of the covariates are presented in S9 Table. (DOCX) [file pone.0210733.s016.docx]

S12 Table. Conditional logit estimates of model “+ prior crime” (Figure 4). Descriptive statistics of the covariates are presented in S9 Table.

| Variable | OR | 95% C.I. | p |
| --- | --- | --- | --- |
| Activity space (16–96] hours | 126.79 | 54.96–292.46 | < .001 |
| Activity space (4–16] hours | 90.61 | 22.92–358.21 | < .001 |
| Activity space (1–4] hours | 50.97 | 19.67–132.08 | < .001 |
| Near activity (1^st^ order) | 31.78 | 19.17–52.71 | < .001 |
| Near activity (2^nd^ order) | 22.92 | 13.20–39.78 | < .001 |
| Near activity (3^rd^ order) | 5.03 | 2.35–10.76 | < .001 |
| Near activity (4^th^ order) | 4.80 | 2.20–10.46 | < .001 |
| Near activity (5^th^ order) | 6.38 | 3.43–11.87 | < .001 |
| Prior crime | 127.95 | 57.60–284.25 | < .001 |
| Near prior crime (1^st^ order) |  |  |  |
| Near prior crime (2^nd^ order) |  |  |  |
| Near prior crime (3^rd^ order) |  |  |  |
| Near prior crime (4^th^ order) |  |  |  |
| Near prior crime (5^th^ order) |  |  |  |
| Retail business |  |  |  |
| Catering business |  |  |  |
| School |  |  |  |
| Crimes | 165 |  |  |
| Locations | 4558 |  |  |
| Accuracy | .84 |  |  |
| McFadden Pseudo R^2^ | .22 |  |  |
