# Supplementary material for: Adolescent offenders' current whereabouts predict locations of their future crimes
Source: PLoS One. 2019 Jan 30;14(1):e0210733. doi: 10.1371/journal.pone.0210733 (PMC6353130; doi:10.1371/journal.pone.0210733)
Supplement: S13 Table — Descriptive statistics of the covariates are presented in S9 Table. (DOCX) [file pone.0210733.s017.docx]

S13 Table. Conditional logit estimates of model “+ prior crime exploration” (Figure 4). Descriptive statistics of the covariates are presented in S9 Table.

| Variable | OR | 95% C.I. | p |
| --- | --- | --- | --- |
| Activity space (16–96] hours | 115.04 | 50.53–261.88 | < .001 |
| Activity space (4–16] hours | 80.41 | 20.89–309.54 | < .001 |
| Activity space (1–4] hours | 48.37 | 18.79–124.52 | < .001 |
| Near activity (1^st^ order) | 25.65 | 15.17–43.36 | < .001 |
| Near activity (2^nd^ order) | 20.35 | 11.90–34.81 | < .001 |
| Near activity (3^rd^ order) | 4.70 | 2.22–9.93 | < .001 |
| Near activity (4^th^ order) | 4.45 | 2.14–9.21 | < .001 |
| Near activity (5^th^ order) | 6.17 | 3.40–11.18 | < .001 |
| Prior crime | 111.63 | 44.45–280.39 | < .001 |
| Near prior crime (1^st^ order) | 3.96 | 1.67–9.43 | 0.002 |
| Near prior crime (2^nd^ order) | 5.48 | 2.39–12.55 | < .001 |
| Near prior crime (3^rd^ order) | 2.50 | 1.24–5.06 | 0.011 |
| Near prior crime (4^th^ order) | 1.67 | 0.59–4.76 | 0.335 |
| Near prior crime (5^th^ order) | 0.79 | 0.29–2.12 | 0.636 |
| Retail business |  |  |  |
| Catering business |  |  |  |
| School |  |  |  |
| Crimes | 165 |  |  |
| Locations | 4558 |  |  |
| Accuracy | .87 |  |  |
| McFadden Pseudo R^2^ | .23 |  |  |
