# Supplementary material for: Adolescent offenders' current whereabouts predict locations of their future crimes
Source: PLoS One. 2019 Jan 30;14(1):e0210733. doi: 10.1371/journal.pone.0210733 (PMC6353130; doi:10.1371/journal.pone.0210733)
Supplement: S14 Table — Descriptive statistics of the covariates are presented in S9 Table. (DOCX) [file pone.0210733.s018.docx]

S14 Table. Conditional logit estimates of model “+ opportunity” (Figure 3 and Figure 4). Descriptive statistics of the covariates are presented in S9 Table.

| Variable | OR | 95% C.I. | p |
| --- | --- | --- | --- |
| Activity space (16–96] hours | 85.01 | 34.47–209.65 | < .001 |
| Activity space (4–16] hours | 51.80 | 12.80–209.59 | < .001 |
| Activity space (1–4] hours | 23.97 | 8.85–64.90 | < .001 |
| Near activity (1^st^ order) | 15.74 | 8.42–29.41 | < .001 |
| Near activity (2^nd^ order) | 13.07 | 7.19–23.77 | < .001 |
| Near activity (3^rd^ order) | 3.14 | 1.41–7.01 | 0.005 |
| Near activity (4^th^ order) | 3.13 | 1.49–6.59 | 0.003 |
| Near activity (5^th^ order) | 4.45 | 2.44–8.12 | < .001 |
| Prior crime | 99.59 | 40.24–246.46 | < .001 |
| Near prior crime (1^st^ order) | 3.87 | 1.61–9.30 | 0.002 |
| Near prior crime (2^nd^ order) | 5.22 | 2.42–11.27 | < .001 |
| Near prior crime (3^rd^ order) | 2.44 | 1.17–5.12 | 0.018 |
| Near prior crime (4^th^ order) | 1.62 | 0.61–4.32 | 0.337 |
| Near prior crime (5^th^ order) | 0.71 | 0.28–1.77 | 0.461 |
| Retail business | 2.00 | 1.37–2.93 | < .001 |
| Catering business | 1.69 | 1.09–2.61 | 0.019 |
| School | 2.94 | 1.64–5.29 | < .001 |
| Crimes | 165 |  |  |
| Locations | 4558 |  |  |
| Accuracy | .95 |  |  |
| McFadden Pseudo R^2^ | .25 |  |  |
