# Supplementary material for: Adolescent offenders' current whereabouts predict locations of their future crimes
Source: PLoS One. 2019 Jan 30;14(1):e0210733. doi: 10.1371/journal.pone.0210733 (PMC6353130; doi:10.1371/journal.pone.0210733)
Supplement: S15 Table — Distance from activity space and distance from prior crime are measured as the contiguity order of a grid cell, with a maximum of 7 for any grid cells beyond 6th order contiguity, and 0 for grid cells inside activity space and grid cells were the offender committed a prior offense, respectively. (DOCX) [file pone.0210733.s019.docx]

S15 Table. Conditional logit estimates of a parsimonious version of the model “+ opportunity” Distance from activity space and distance from prior crime are measured as the contiguity order of a grid cell, with a maximum of 7 for any grid cells beyond 6^th^ order contiguity, and 0 for grid cells inside activity space and grid cells where the offender committed a prior offense, respectively.

| Variable | OR | 95% C.I. | p |
| --- | --- | --- | --- |
| Number of hours activity space (0-96) | 1.02 | 1.00-1.04 | 0.026 |
| Distance from activity space (per 200m) | 0.60 | 0.54-0.68 | < .001 |
| Distance from prior crime (per 200m) | 0.42 | 0.36-0.49 | < .001 |
| Retail business | 1.94 | 1.29-2.92 | 0.001 |
| Catering business | 1.7 | 1.11-2.60 | 0.015 |
| School | 2.71 | 1.43-5.13 | 0.002 |
| Crimes | 165 |  |  |
| Locations | 4558 |  |  |
| Accuracy | .94 |  |  |
| McFadden Pseudo R^2^ | .25 |  |  |
